# Bacteriological and physicochemical quality assessment of municipal solid waste landfill in the fragile ecosystem of Eastern Himalayan region: a study from Martam, East Sikkim

**DOI:** 10.1007/s10661-026-15068-9

**Published:** 2026-02-23

**Authors:** Priyanka Chettri, Dechen Chomu Tsering, Sudarsan Tamang

**Affiliations:** 1 Department of Microbiology, Sikkim Manipal Institute of Medical Sciences, Sikkim Manipal University, Gangtok, 737102 Sikkim India; 2 Department of Chemistry, School of Physical Sciences, Sikkim University, Tadong, Gangtok, 737102 Sikkim India

**Keywords:** Bioremediation, Integrated waste management, Landfill soil, Leachate impact, Physicochemical characteristics, Waste deposition

## Abstract

Rapid urbanization has intensified waste management challenges in the fragile Eastern Himalayan region, especially in the rapidly urbanizing state of Sikkim. To assess landfill impacts on soil health, we analyzed physicochemical and microbial characteristics of soils from the Martam landfill, Sikkim, against a control, for comparative analysis. Landfill soils showed neutral–alkaline pH (7.38–8.02), elevated moisture, phosphorus, and potassium, but depleted organic carbon and nitrogen, indicating nutrient imbalance and soil’s organic component loss. Microbial profiling revealed 40 isolates, dominated by *Bacillus* and *Pseudomonas* spp. bacteria with known plastic-degrading potential alongside halophilic *Salinicoccus roseus*, indicative of chemical stress from leachates. These findings demonstrate that landfill accumulation alters soil ecology but also harbours microbial candidates for biodegradation. Understanding these parameters is essential for developing sustainable waste management and soil remediation strategies. For land-scarce regions like Sikkim, reducing landfill reliance through integrated waste management and sustainable treatment technologies is critical for ecological resilience.

## Introduction

In 2023, India officially overtook China to become the most populous country in the world, marking a historic demographic milestone (Srinivasan & Srinivasan, 2023; Hertog et al., 2023). With the global population currently around 7.3 billion and projected to rise to nearly 9 billion in the coming decades (Mustaq et al., 2020), the challenges surrounding urbanization, consumption, and waste generation are growing at an alarming rate. Rapid urban expansion and intensifying consumer lifestyles have dramatically increased both the volume and complexity of municipal solid waste (MSW), leading to mounting environmental and public health concerns.

It is estimated that global MSW generation currently stands at approximately 2.01 billion tons annually, of which 33% is not managed in an environmentally sound manner (Mustaq et al., 2020). If current trends persist, global waste is expected to rise to 3.4 billion tons by 2050 (Kaza et al., 2018). In India alone, daily MSW generation exceeds 168,000 tons and is projected to increase significantly as the country’s population may reach 2.6 billion by 2051 (Mustaq et al., 2020). In this context, effective and sustainable waste management has become one of the most urgent challenges faced by developing nations. The continued use of inadequate practices—such as open dumping, landfilling, and incineration—particularly in low-income countries, has exacerbated pollution and environmental degradation (Ibe et al., 2020; Iheanacho et al., 2022). Landfilling remains the most widely used, cost-effective waste disposal method, despite its long-term ecological consequences (Oziegbe et al., 2021).

In India’s Eastern Himalayan region—an ecologically diverse and geologically fragile zone—the waste management challenge is further compounded by unique topographical and infrastructural constraints. The Indian Himalayan Region (IHR), home to nearly 50 million people, is experiencing rapid development, fueled in part by the tourism industry (Aayog, 2018; Gupta et al., 2019; Thakur et al., 2021). However, this development has come at the cost of increasing solid waste generation, placing a significant burden on the region’s already limited waste management infrastructure. The scarcity of land for landfill construction, particularly in mountainous terrains, has intensified this problem (Rai et al., 2024).

Sikkim, covering an area of 7096 sq. km, is one of the hilly states in India. Nesting amidst the Eastern Himalayas, it is located between 27°17′20″ to 27°21′47″ N and 88°35′12″ to 88°39′40″ E (Rai & Singh, 2022). Sikkim has experienced rapid growth and modernization since its merger with India in 1975. This transition has led to a noticeable conversion from a sustainable farming-based economy to a service-driven one, particularly in the city of Gangtok which is the capital of Sikkim. This transformation reflects broader economic changes, impacting the state’s socio-economic landscape and environmental sustainability (Indira, 2021).

Waste generation and management has become a major challenge in Sikkim recently. Most waste in Sikkim originates from domestic, medical, commercial, and sanitary sources (Indira, 2021). The state generates around 71.9 TPD (tonnes per day) of solid waste, out of which 20.35 TPD is treated and the rest is landfilled or dumped. This means approximately 28.3% of the waste is treated, while 71.7% is landfilled or dumped (Rai et al., 2024). According to the Annual Report on solid waste management for the year 2022–2023, the 7 major urban local bodies generate 68.9 TPD of solid waste, all of which is collected; 23.7 TPD is treated through composting and recycling whereas 45.2 TPD is landfilled/disposed at dumping sites (Sikkim Pollution Control Board, 2023). Sikkim has a population of 6,10,577 (according to the 2011 census), with a density of 86 persons km^−2^, whereas Gangtok exceeds 7000 persons km^−2^ (Indira, 2021). The Gangtok Municipal Corporation (GMC) manages urban planning and municipal services, with a reported MSW generation of 50 TPD comprising 57% of which is biodegradable and 43% non-biodegradable waste (Indira, 2021).

Despite being one of India’s most environmentally progressive states, Sikkim is now grappling with mounting waste-related challenges. Cities like Gangtok rely on landfill sites situated perilously close to riverbanks, such as the Martam landfill. These sites pose serious environmental threats, contaminating both soil and nearby water bodies. Leachate and runoff from these landfills feed into rivers like the Teesta, which flows downstream through West Bengal and into the Bay of Bengal, thereby threatening ecosystems, agriculture, and human health across regions. Moreover, logistical challenges such as steep, narrow roads in this hilly terrain impede timely waste collection and transportation, often resulting in open dumping and waste burning (Rai et al., 2024).

These challenges highlight the urgent need for sustainable and region-specific waste management policies. Inadequate segregation, co-disposal of hazardous and recyclable waste, and poorly designed landfills continue to be key contributors to environmental degradation (Kebede et al., 2016). Without a strategic shift toward sustainable practices—such as reducing waste at source, improving recycling systems, and minimizing reliance on traditional landfilling—the growing volume of solid waste may overwhelm infrastructure and further strain the environment.

In this study, physicochemical analyses were coupled with bacteriological studies to provide a comprehensive understanding of the landfill soil environment. While physicochemical parameters indicate nutrient status, pH, and contamination levels, bacterial profiling reveals the microbial diversity—including potential plastic-degrading and halotolerant species—and potential for natural biodegradation, allowing us to correlate soil conditions with microbial community structure and ecological functionality. Bacteriological assessment is particularly important, as bacteria are the primary drivers of waste decomposition. Landfill environments host distinct and functionally important bacterial communities that directly influence waste degradation rates (Ke et al., 2022; Stamps et al., 2016). Functional prediction and substrate utilization analyses further indicate that dumpsite soils harbour active bacterial communities with strong degradative capabilities (Selvarajan et al., 2022). The study seeks to understand how ongoing landfill operations are affecting soil health and the broader environment. The results are expected to offer valuable insights into the ecological consequences of unmanaged waste disposal and serve as a foundation for improved waste management and remediation strategies in this sensitive Himalayan ecosystem. In doing so, the study contributes toward building a scientific basis for regionally tailored, ecologically responsible waste management solutions.

## Materials and methods

### Study area

**Fig. 1:**
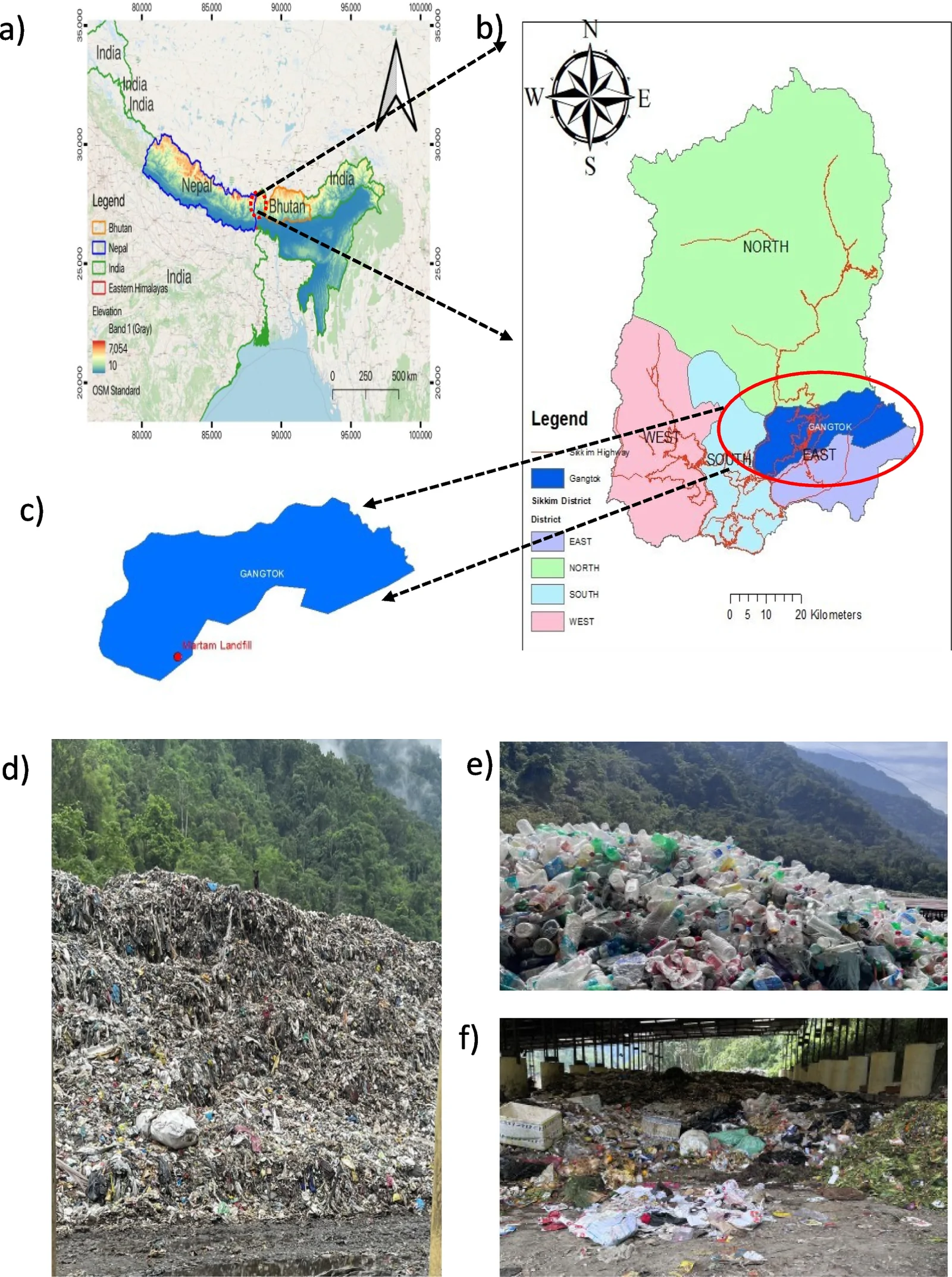
(**a**) A portion of the map showing the Eastern Himalayan belt where Sikkim is located. (**b**) Map of Sikkim and (**c**) location of the Martam landfill under study. (**d–f**) are representative photographs of the landfill. All samples were collected from open sites

Soil sample was collected from Martam landfill (Fig. 1). The waste dump site is located at Lower Martam areas of East Sikkim, 20 km away from Gangtok and is located adjacent to NH 31A. The plot is precisely located at 27°15′30.6″ N and 88°33′04.5″ E. Landfill area is 10,970 sq. m (1.1 ha). Approximate dimensions of site are 140 m width and 340 m length (Government of Sikkim State investment Programme Management and Implementation Unit, SIPMIU, 2011). Additionally, Martam landfill consists of composting facilities and machinery (Rai et al., 2024).

### Sample collection

**Fig. 2:**
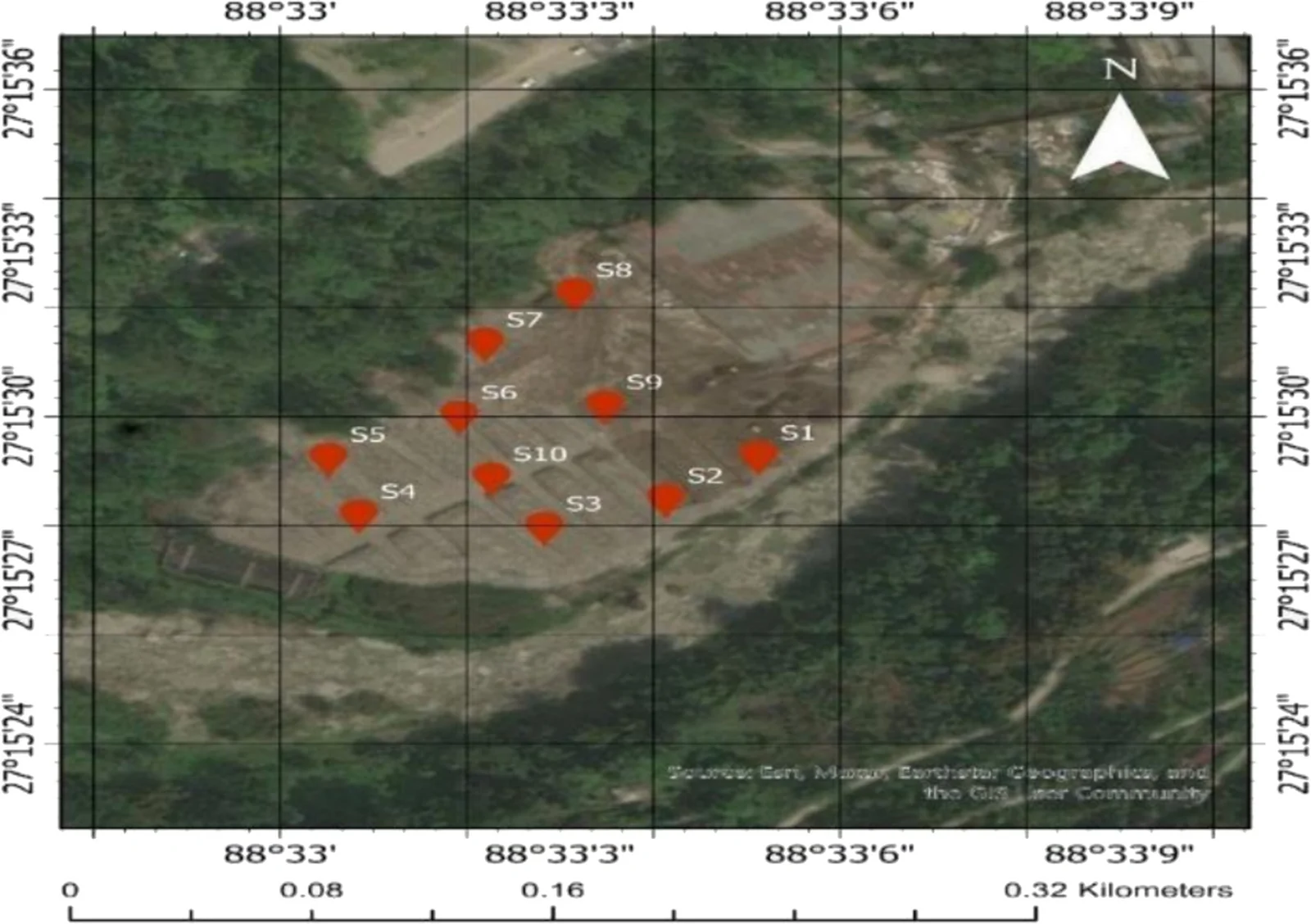
Sampling sites across the Martam landfill

Ten-centimeter deep soil samples were collected from the open landfill sites (Oziegbe et al., 2021). The locations of the sampling sites are shown in Fig. 2. During sampling, the debris was carefully removed from the surface of the sampling point, subsurface scooped at 10 cm depth, and transferred into sterile zip lock bags. Since the Martam landfill sits along the banks of the Ranikhola River, the soil found there is sandy in nature. The as-collected crude soil samples were air-dried and sorted to remove nondegradable materials: stones, bottles, and plastics. The resultant samples were aseptically ground and sieved (Essien et al., 2011). The processed MSW powder was used for subsequent microbiological and physicochemical analyses (Emmanuel et al., 2017; Essien et al., 2011). The samples were collected during the month of March 2023 and were immediately dried and analyzed to ensure the reliability of the analyses. A control soil sample was collected from an adjacent undisturbed site approximately 100 m from the study area, as this site provided a suitable reference closely matching the soil type, vegetation, and land-use characteristics of the study area.

### Isolation of bacteria from waste samples

Isolation of bacteria were performed by serial dilution plate technique using nutrient agar media (Essien et al., 2011). One gram of soil was added to 10 ml of sterile water (the stock). Serial dilution was performed from the stock by transferring 1 ml into sterile 9 ml diluent creating 10^–1^ dilution (Saha & Santra, 2014). One milliliter from 10^–1^ dilution was then transferred to the 10^–2^ containing the same volume of sterile diluent to create 10^–2^ dilution. The dilution and mixing process was repeated for each subsequent dilution blank up to 10^–8^ dilution (Saha & Santra, 2014). One milliliter was taken from different dilutions and inoculated into a sterile Petri dish (Emmanuel et al., 2017). Twenty milliliters of sterile nutrient agar was then poured, swirled, and allowed to solidify. The plates were then incubated at 35 °C for 2 days (Emmanuel et al., 2017).

### Characterization and identification of the bacterial isolates

The isolated bacterial colonies specially the unique ones were sub-cultured on fresh nutrient agar plates and subsequently incubated at 35 °C for 24 h (Deshmukh & Aher, 2017).

The sub-cultured bacterial colonies were identified using the standard morphological and biochemical identification method (Emmanuel et al., 2017; Iheanacho et al., 2022). Isolated bacteria were further cultured on nutrient agar slants and stored at 4 °C.

### Physicochemical analyses of the soil samples

#### Moisture content (%) and pH determination

Initial weight of the freshly collected sample was recorded and then kept inside a hot air incubator at 110 °C. The sample was then weighed several times. Finally, the constant weight achieved after drying was recorded. The following formula was used to calculate soil moisture content (Saha & Santra, 2014):

$$MC\%=\frac{(W-w)}{w}\times 100$$

where *MC* is moisture content, *W* is the original weight, and *w* is the constant weight after oven drying.

The pH was determined with the help of a calibrated pH meter (LABMAN) in a 1:2.5 soil–water mixture. Air-dried and sieved soil was mixed with distilled water at a ratio of 1:2.5 (w/v), shaken thoroughly, and allowed to equilibrate before measurement (Mouhoun-Chouaki et al., 2019; Saha & Santra, 2014).

#### Chemical analysis of waste

The total organic carbon (TOC) of the soil sample was estimated using standard Walkey method (Walkley & Black, 1934)*.* Kjeldahl method was used to determine the total nitrogen (N) content of the soil samples. Total phosphorus (P) was measured by colorimetric method and potassium (K) content was deduced using flame photometric method (Osazee et al., 2013; Saha & Santra, 2014).

### Statistical analysis

Descriptive statistical analysis was performed for the obtained data including the calculation of mean, standard deviation (SD), range, and coefficient of variation (CV). Higher-order statistical measures such as skewness and kurtosis were also assessed to evaluate data distribution. Correlation analysis was performed to examine relationships among six physicochemical parameters (pH, moisture content, total organic carbon, total nitrogen, available phosphorus, and potassium). Additionally, hierarchical cluster analysis (HCA) was applied for patterns and groupings among soil samples based on their physicochemical characteristics.

## Results and discussion

Landfills host diverse communities of microorganisms, and the heterogeneous nature of municipal waste makes them one of the most dynamic terrestrial ecosystems (Stamps et al., 2016). The current investigation was carried out to obtain an insight about microbial communities (mainly bacteria) present in the waste dump site. A total of 40 bacterial strains were isolated. Morphological and microscopic observations were done to identify the bacterial isolates (Tables 1, 2, and 3). The colony characteristics and gram nature of the bacterial isolates were observed and biochemical identification was done (Saha & Santra, 2014).

**Table 1:** Biochemical test results for gram-positive cocci isolated from Martam landfill

ISOLATES	GRAM’S STAIN	BIOCHEMICAL TESTS														INFERENCE
	Gram positive bacilli	CAT	OXI	IND	CIT	URE	TSI	MR	VP	MOT	NIT	GLU	LAC	SUC	MAN	
ISL 8		+	+	-	-	-	K/A	+	+	+	-	+	-	+	-	*Bacillus pumilis*
ISL 9		+	-	-	+	-	A/A	-	+	+	-	+	-	+	-	*Bacillus cereus*
ISL 11		+	+	-	-	-	K/A	+	+	+	-	+	-	+	-	*Bacillus pumilis*
ISL 13		+	-	-	+	-	K/A	-	+	+	+	+	-	+	+	*Bacillus subtilis*
ISL 14		+	+	-	+	+	A/A	-	+	+	+	+	+	+	+	*Bacillus licheniformis*
ISL 15		+	-	-	+	+	K/K	+	-	+	-	+	+	+	+	*Bacillus megaterium*
ISL 16		+	+	-	+	+	A/A	-	+	+	+	+	+	+	+	*Bacillus licheniformis*
ISL 17		+	-	-	+	-	A/A	-	+	+	-	+	-	+	-	*Bacillus cereus*
ISL 18		+	-	-	+	-	K/A	-	+	+	+	+	-	+	+	*Bacillus subtilis*
ISL 22		+	+	-	-	-	K/A	+	+	+	-	+	-	+	-	*Bacillus pumilis*
ISL 23		+	-	-	+	-	K/A	-	+	+	+	+	-	+	+	*Bacillus subtilis*
ISL 24		+	+	-	+	+	A/A	-	+	+	+	+	+	+	+	*Bacillus licheniformis*
ISL 25		+	-	-	+	-	K/A	-	+	+	+	+	-	+	+	*Bacillus subtilis*
ISL 26		+	+	-	-	-	K/A	+	+	+	-	+	-	+	-	*Bacillus pumilis*
ISL 28		+	-	-	+	-	K/A	-	+	+	+	+	-	+	+	*Bacillus subtilis*
ISL 33		+	-	-	+	-	A/A	-	+	+	-	+	-	+	-	*Bacillus cereus*
ISL 35		+	+	-	-	-	K/A	+	+	+	-	+	-	+	-	*Bacillus pumilis*

**Table 2:** Biochemical test results for gram-negative bacilli isolated from Martam landfill

Isolates	Gram’s stain	Biochemical tests														Inference
		OF	CAT	OXI	NIT	IND	CIT	TSI	URE	MR	VP	GLU	LAC	SUC	MAN	
ISL 5	Gram-negative bacilli	Oxidative	+	+	−	−	−	K/K	−	−	−	+	−	−	−	*Pseudomonas putida*
ISL 7		Fermentative	+	−	+	+	+	K/A	+	+	−	+	−	+	−	*Proteus vulgaris*
ISL 10		Fermentative	+	−	+	+	−	A/A	−	+	−	+	+	+	+	*E. coli*
ISL 12		Oxidative	+	+	+	−	+	A/A	−	−	−	+	−	−	−	*Pseudomonas aeruginosa*
ISL 19		Oxidative	+	+	−	−	−	K/K	−	−	−	+	−	−	−	*Pseudomonas putida*
ISL 20		Fermentative	+	−	+	+	−	A/A	−	+	−	+	+	+	+	*E. coli*
ISL 21		Non-saccharolytic	+	+	−	−	−	K/A	+	−	−	+	−	−	−	*Pseudomonas alcaligens*
ISL 32		Fermentative	+	−	+	−	+	A/A	+	−	+	+	+	+	+	*Klebsiella pneumoniae*
ISL 36		Fermentative	+	−	+	+	−	A/A	−	+	−	+	+	+	+	*E. coli*
ISL 38		Fermentative	+	−	+	+	−	A/A	−	+	−	+	+	+	+	*E. coli*
ISL 40		Oxidative	+	+	+	−	+	A/A	−	−	−	+	−	−	−	*Pseudomonas aeruginosa*

Legend: +: positive; −: negative; K/A: alkaline slant/acidic butt; A/A: acidic slant/acidic butt; K/K: alkaline slant/alkaline butt

Abbreviations: *CAT*, catalase; *OXI*, oxidase; *IND*, indole; *CIT*, citrate, *URE*, urease; *TSI*, triple sugar iron; *MR*, methyl red; *VP*, Voges-Proskauer; *OF*, oxidative/fermentative; *NIT*, nitrate; *GLU*, glucose; *LAC*, lactose; *SUC*, sucrose; *MAN*, mannitol

**Table 3:** Biochemical test results for gram-positive bacilli isolated from Martam landfill

Isolates	Gram’s stain	Biochemical tests														Inference
ISL 8	Gram-positive bacilli	CAT	OXI	IND	CIT	URE	TSI	MR	VP	MOT	NIT	GLU	LAC	SUC	MAN	
ISL 9		+	+	−	−	−	K/A	+	+	+	−	+	−	+	−	*Bacillus pumilis*
ISL 11		+	−	−	+	−	A/A	−	+	+	−	+	−	+	−	*Bacillus cereus*
ISL 13		+	+	−	−	−	K/A	+	+	+	−	+	−	+	−	*Bacillus pumilis*
ISL 14		+	−	−	+	−	K/A	−	+	+	+	+	−	+	+	*Bacillus subtilis*
ISL 15		+	+	−	+	+	A/A	−	+	+	+	+	+	+	+	*Bacillus licheniformis*
ISL 16		+	−	−	+	+	K/K	+	−	+	−	+	+	+	+	*Bacillus megaterium*
ISL 17		+	+	−	+	+	A/A	−	+	+	+	+	+	+	+	*Bacillus licheniformis*
ISL 18		+	−	−	+	−	A/A	−	+	+	−	+	−	+	−	*Bacillus cereus*
ISL 22		+	−	−	+	−	K/A	−	+	+	+	+	−	+	+	*Bacillus subtilis*
ISL 23		+	+	−	−	−	K/A	+	+	+	−	+	−	+	−	*Bacillus pumilis*
ISL 24		+	−	−	+	−	K/A	−	+	+	+	+	−	+	+	*Bacillus subtilis*
ISL 25		+	+	−	+	+	A/A	−	+	+	+	+	+	+	+	*Bacillus licheniformis*
ISL 26		+	−	−	+	−	K/A	−	+	+	+	+	−	+	+	*Bacillus subtilis*
ISL 28		+	+	−	−	−	K/A	+	+	+	−	+	−	+	−	*Bacillus pumilis*
ISL 33		+	−	−	+	−	K/A	−	+	+	+	+	−	+	+	*Bacillus subtilis*
ISL 35		+	−	−	+	−	A/A	−	+	+	−	+	−	+	−	*Bacillus cereus*

Legend: +: positive; −: negative; K/A: alkaline slant/acidic butt; A/A: acidic slant/acidic butt; K/K: alkaline slant/alkaline butt

Abbreviations: *CAT*, catalase; *OXI*, oxidase; *IND*, indole; *CIT*, citrate, *URE*, urease; *TSI*, triple sugar Iron; *MR*, methyl red; *VP*, Voges-Proskauer; *MOT*, motility; *NIT*, nitrate; *GLU*, glucose; *LAC*, lactose; *SUC*, sucrose; *MAN*, mannitol

**Fig. 3:**
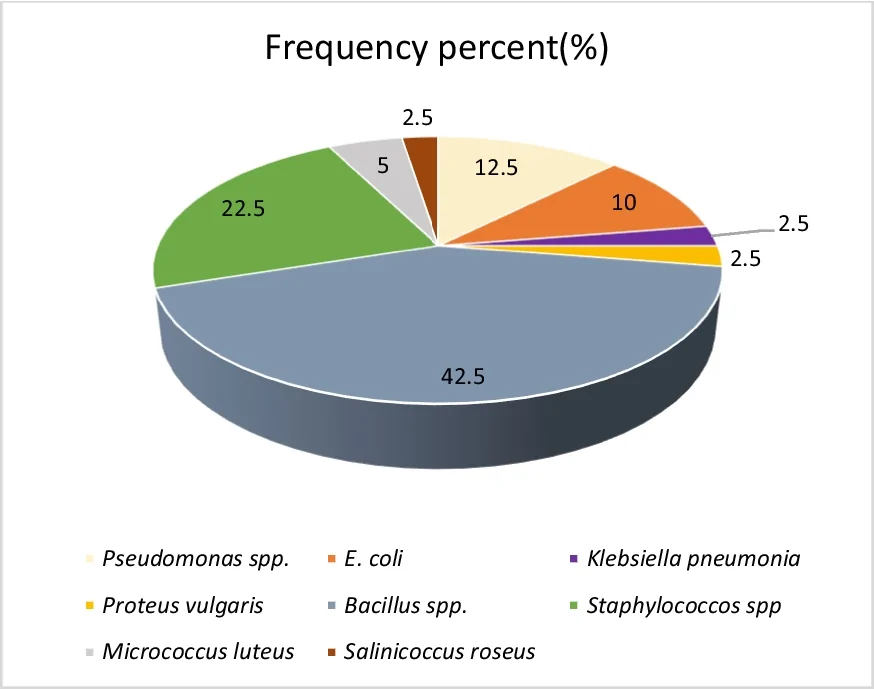
Percentage frequency of occurrence of the microbial isolates from the landfill

We observed that the bacterial isolates from the MSW predominantly consisted of gram-positive bacteria that included *Staphylococcus* spp., coagulase-negative *Staphylococcus* spp. (CONS), and *Bacillus* spp. with *Bacillus* spp. exhibiting the highest frequency of isolation (Fig. 3). *Micrococcus luteus* and *Salinicoccus roseus* had the least frequency of isolation. Several research articles have investigated the microbial communities in landfill environments and have found that gram-positive bacteria, particularly those belonging to the phylum Firmicutes are prevalent (Selvarajan et al., 2022; Song et al., 2015). These bacteria possess thick peptidoglycan cell walls, which may contribute to their dominance in such harsh environments. A study on the microbial diversity in landfill soil from Gorakhpur, India, identified gram-positive bacilli as predominant strains, suggesting their significant role in the landfill’s microbial composition (Singh & Srivastava, 2024). Additionally, research on landfilled MSW in China reported that Firmicutes, known for their hydrolytic and fermentative capabilities, were among the main phyla present highlighting their importance in the degradation of complex organic materials within landfills (Ke et al., 2022). Krishnamurthi and Chakrabarti (2013) also reported similar findings and identified *Bacillus* as the predominant genus among fermentative and hydrolytic bacteria. Firmicutes are known for their cellulose-degrading capabilities playing a crucial role in the anaerobic and methanogenic phases of refuse decomposition in landfills (Wang et al., 2017). Their dominance suggests both the adaptability and functional significance of gram-positive bacteria in landfill ecosystem, as well as the presence of high cellulose-rich organic matter in such environments.

Gram-negative bacteria consisted mainly of *Pseudomonas* spp. *and E. coli*. Other least occurring gram-negative bacteria were found to be *Klebsiella* sp. and *Proteus* sp. Gram-negative bacteria possess inherent resilience due to their protective outer membrane, versatile metabolism, and stress response mechanisms which allow them to survive under a variety of environmental stresses (Castro et al., 2025). Despite this, the specific conditions at our study site appear to favour the proliferation of Gram-positive taxa, suggesting that environmental pressures, rather than intrinsic cellular resistance alone, play a key role in shaping the observed microbial community composition. Additionally, Gram-positive bacteria can secrete enzymes to break down complex organic matter enabling them to utilize a wider range of carbon sources, whereas Gram-negative bacteria rely on transport proteins and prefer simpler nutrients (Sekhohola-Dlamini et al., 2021). These traits likely contribute to the higher prevalence of Gram-positive bacteria in our study.

The frequency of occurrence of the isolated bacteria from landfill was as follows (Table 1): *Pseudomonas* sp. (12.5%), *E. coli* (10%), *Klebsiella* sp. (2.5%), *Proteus* sp. (2.5%), *Bacillus* sp. (42.5%), *Staphylococcus* sp. (22.5%), *Micrococcus* sp. (5%), *Salinicoccus* sp. (2.5%). All of the bacterial isolates are commonly observed microorganisms in the landfill (Essien et al., 2011) except for *Salinicoccus roseus*, which is a known halophile, thriving in high salt environment (Dutta et al., 2023). *Salinicoccus roseus* is an obligate aerobe demonstrating optimal growth at NaCl concentrations ranging from 5 to 15%, with some strains tolerating up to 25% NaCl. This bacterium grows within a temperature range of 15 to 45 °C and pH between 6 and 9 (Javed et al., 2023). The isolation of *Salinicoccus roseus* from solid waste landfill may suggest the presence of saline conditions or the influence of landfill leachates containing salts or other compounds conducive to halophilic microbial growth. This discovery also suggests unique adaptability or survival mechanisms of *Salinicoccus roseus* broadening our understanding of where halophiles can survive. Among the other microorganisms isolated, *Staphylococcus* also exhibited a high frequency, which is consistent with findings of Obire et al. (2002). The occurrence of *Bacillus *spp., *Pseudomonas* spp., and *Micrococcus* spp. in the municipal waste dumpsite has also been reported by Emmanuel et al. (2017). A high percentage of *Bacillus* spp., followed by *Micrococcus* spp., and *E. coli* were isolated from open waste dumpsites of the Kathmandu city, Nepal (Kapali et al., 2023). *Bacillus subtilis* and *Pseudomonas aeruginosa* possess a strong capability for waste degradation (Essien et al., 2011). Previous studies have reported that *Bacillus* and *Pseudomonas* species are capable of degrading low-density polyethylene (Wanapat et al., 2025; Yao et al., 2022). The association of all the identified microbial isolates from soil samples with waste and waste biodegradation has been previously reported (Essien et al., 2011).

Most of the above isolated bacterial species have been implicated as opportunistic human pathogens (Srivastava et al., 2020).

### Physicochemical analyses

Physicochemical analysis of soil helps us to understand the native environment of bacteria. The result of physicochemical properties of the soil samples is presented in Table 4. A total of 10 soil samples were collected from Martam landfill. The findings in Table 4 revealed that there was an increase in the physicochemical properties of soil from the landfill than the control site with the exception of total nitrogen. This increase in the soil properties like moisture content, total organic carbon content, phosphorus content, and potassium content of the landfill soil may be due to the breakdown of organic waste and infiltration of leachate (Islam et al., 2024; Parameswari et al., 2015; Regmi et al., 2022).

**Table 4:** Physicochemical properties of Martam landfill soil collected from various locations

Soil samples	pH	Moisture content (%)	Total organic carbon (%)	Total nitrogen (kg ha^−1^)	Available phosphorus (kg ha^−1^)	Potassium (kg ha^−1^)
S1	7.74	30.27	2.59	87.8	13.21	1052.8
S2	7.87	26.62	1.3	31.36	8.04	1008
S3	7.72	28.20	2.46	47.04	21.05	1064
S4	7.38	22.35	1.27	94.08	18.48	1456
S5	7.71	20.35	1.09	94.08	13.21	1388.8
S6	8.02	27.27	2.79	25.08	18.48	1993.6
S7	7.95	28.60	3.18	47.04	23.63	1612.8
S8	7.84	21.94	2.27	62.72	8.064	1209.6
S9	7.52	26.27	1.56	47.04	13.21	1545.6
S10	7.67	18.02	0.88	62.72	8.064	1052.8
**Control**	**8.74**	**18.15**	**2.09**	**213.24**	**11.2**	**313.6**
Mean	7.74	24.9	1.9	59.89	14.54	1338.4
Standard deviation	0.19	4.04	0.81	25.06	5.65	321.12
Range	0.64	12.25	2.3	69	15.59	985.6
Co-efficient of variance	2.45478	16.2249	42.63158	41.84338	38.85832	23.99283
Skewness	−0.492	−0.503	0.148	0.263	0.247	0.869
Kurtosis	0.083	−1.032	−1.644	−1.256	−1.256	0.241

### pH measurement

**Fig. 4:**
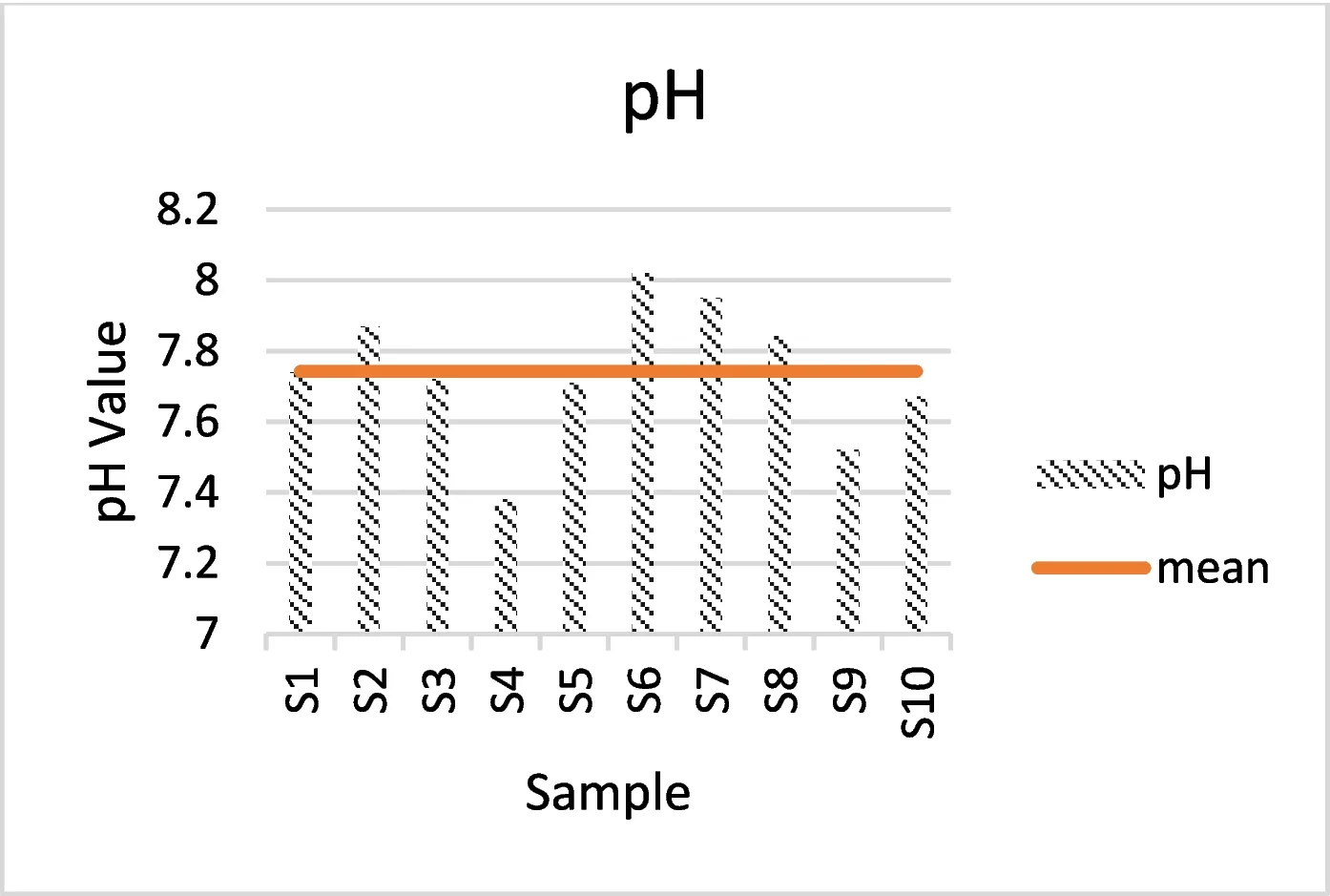
pH values of 10 soil samples collected from Martam landfill. The figure displays individual values and the mean, indicating mostly basic soil conditions across the sampling sites

The soil samples analyzed were mostly of neutral pH, with values ranging from 7.38 to 8.02, the mean of 7.74 (Fig. 4) and coefficient of variance (CV) of 2.45% (Table 4), indicating low variability. The control site had mean pH value of 8.74. pH of the soil is an important parameter that affects the flow of heavy metals in soil. Nutrient availability is also dependent on soil pH. Metallic elements solubility increases with the decrease in pH, making them more readily available to plants. Lower pH levels, however, favour the availability of the metals like lead and cadmium in soil due to increased solubility of these ions in acidic environments (Top et al., 2019). Consequently, soils having acidic pH levels are rendered unsuitable for waste land filling (Emmanuel et al., 2017). A pH between 6 and 8 is highly favourable for microbial communities typically responsible for decomposition in landfill and soil environments. While this range is considered optimum, it is important to note that a significant drop in pH can slow decomposition because overall microbial diversity and activity decline. On the other hand, if the pH reaches to 9, nitrogen is converted to ammonia making it unavailable for organisms, ultimately causing the process of decomposition of solid waste to slow down (Top et al., 2019). A similar observation was reported by Saha and Santra (2014), where the pH value of 7.79 was found in soil samples of Barrackpore Municipality dumpsite in West Bengal. Cai et al. (2024), also reported the soils around landfill were from neutral to weak alkalinity with pH ranging from 7.29 to 7.91. Akintola et al. (2021) found pH values of 6.68–88.88 (mean 7.85), while Chouaki et al. (2019) reported a neutral range of 6.95–7.4, and Ogundiran et al. (2008) recorded a mean of 7.88 ± 0.25. In contrast, Bella-Osagie and Ekhaise (2017) observed a more acidic pH levels (5.2–6.9), associated with greater micronutrient solubility and mobility as well as higher concentrations of heavy metals.

### Moisture content (MC) determination

**Fig. 5:**
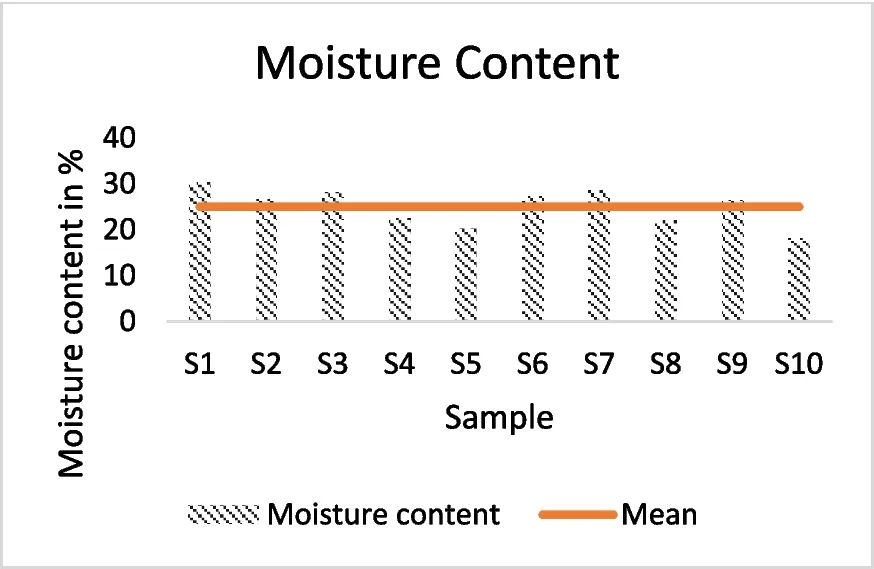
Moisture content of 10 soil samples from Martam landfill. Mean value is shown to highlight general soil water retention capacity

Moisture (water) is important in the breakdown of contaminants because it affects the kind and amount of soluble materials available. Water is required by microorganisms for nutrient assimilation, metabolism, and reproduction. Hence, moisture content affects microbial activity and thus affects the rate of decomposition (Top et al., 2019). The moisture content is pivotal in comprehending the characteristics of waste. High moisture content suggests high organic matter in waste (Rana et al., 2017). In the present investigation, the moisture content in the dumpsite was found ranging between 18.02 and 30.27%, with a mean value being 24.9% (Fig. 5) and CV being 16.22%, suggesting moderate variability in water retention. This variation in the data reflects heterogeneity in water availability, potentially affecting microbial activity and organic matter breakdown. Slightly lower moisture content (18.15%) was recorded from the control site soil sample. The higher moisture content in the landfill soil may be due to water retention from mixed waste and leachates which also contribute to high organic matter (Islam et al., 2024). Studies conducted in Chandigarh, Mohali, and Panchkula showed moisture content ranging from 42 to 59%, 34 to 57.7%, and 35 to 44% respectively (Rana et al., 2017). Tripathi and Misra (2012) reported good amounts of moisture content (35.53 ± 1.79 to 50.77 ± 1.32%) from dumpsites in Allahabad. Barrackpore Municipality and Dhapa waste dumpsite in West Bengal also recorded high moisture content of 65.32% and 66.45% respectively (Saha & Santra, 2014). In contrast, a low moisture content around Vellalore disposal site was reported ranging from 6 to 7.14% in 2012 and from 6 to 7.12% in 2013 (Uma et al., 2016).

### Chemical analysis of waste

#### Organic carbon

**Fig. 6:**
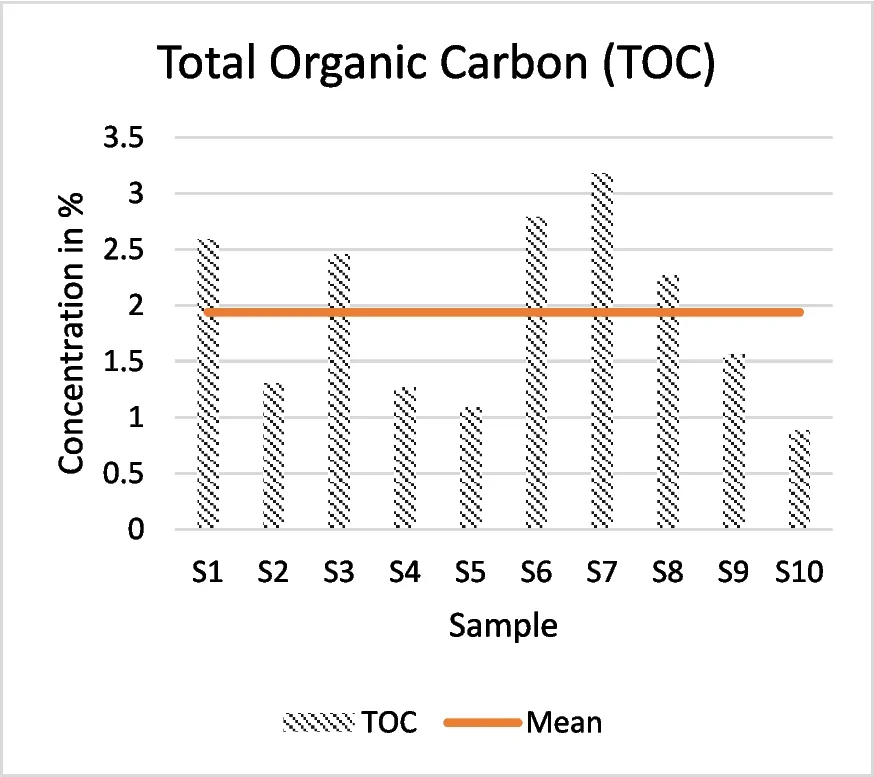
Total organic carbon (TOC) content in 10 soil samples. The data shows organic carbon variation with a mean TOC value indicated

Total carbon content of the soil samples was found to be moderate ranging from 0.88 to 3.18%, the mean TOC (total organic carbon) value being 1.9 ± 0.81% (Fig. 6) and exhibiting high variability with CV of 42.63%. TOC content of the control site was slightly higher (2.09%). Amos-Tautua et al. (2014) also presented similar findings. In the study conducted by Iheanacho et al. (2022), the percentage of TOC obtained, however, ranged from 3.92 ± 1.80 to 9.50 ± 3.00. A high percentage of organic carbon ranging from 0.8 to 12.2% was reported by Deshmukh and Aher (2017). In contrast, the findings reported by Ulakpa et al. (2021) showed that the organic carbon content of the dumpsite soil ranged from 0.05 ± 0.00 to 0.64 ± 0.03%. This finding of low organic carbon content in the dumpsite soil by Ulakpa et al. (2021) was associated with high sand content of the soil. Due to the proximity of Martam landfill to the river, the soil in the area has higher sand content, which may be the reason for lower organic carbon content in the soil. Organic carbon present in the soil increases the cation exchange capacity which enhances the process of nutrient assimilation by plants. As a major food source for microbes, it also influences microbial activity (Amos-Tautua et al., 2014).

#### Total nitrogen (N)

**Fig. 7:**
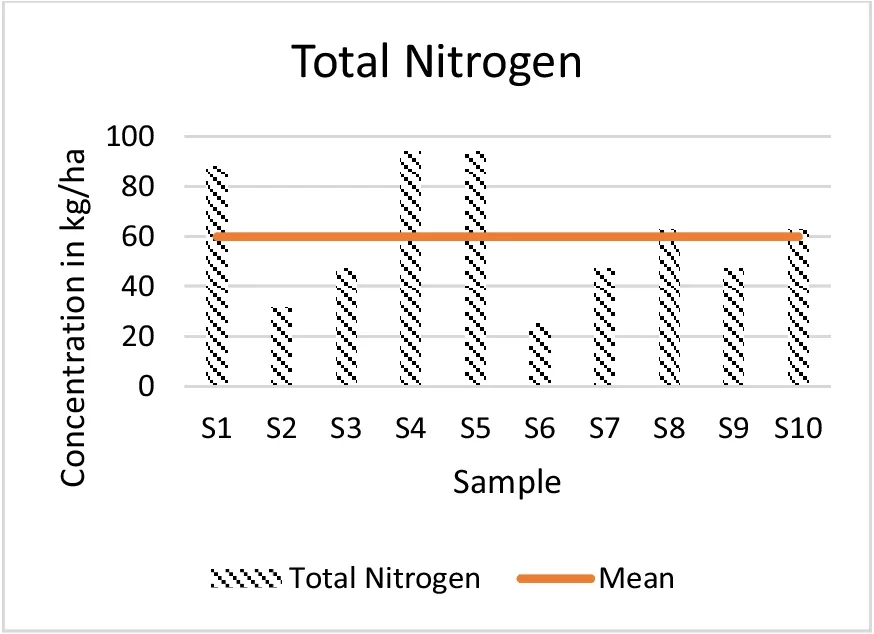
Nitrogen concentrations in 10 soil samples. The figure includes individual data points and the mean value showing nutrient availability in the soil

The data pertaining to the mean and range of total nitrogen in soil samples collected from the Martam landfill is presented in Table 4. The findings showed extremely low levels of total nitrogen in the soil samples collected from different regions of the dumpsite ranging from 25.08 to 94.08 kg ha^−1^, the mean value being 59.89 kg ha^−1^ (Fig. 7). The coefficient of variation of nitrogen was 41.84%, indicating high variability among the samples. The control site had higher levels of nitrogen (213.24 kg ha^−1^). This phenomenon is in contrast with Ulakpa et al. (2021) and Regmi et al. (2022), who reported higher nitrogen contents of 0.93 ± 0.03 to 0.39 ± 0.01 and 119.7 kg ha^−1^, respectively. The soil samples around the dumpsites of Ghana also recorded low concentrations of nitrogen, 0.09 ± 0.1 to 0.897 ± 1.2% (Gyabaah et al., 2023). Similarly, total nitrogen content of 1.35 ± 0.04 g kg^−1^ was reported by Cai et al. (2024). However, all the studies reported higher nitrogen content in landfill soil in comparison to the control sample. In contrast, we observed higher nitrogen content in control soil than landfill soils. Such low levels of nitrogen can be attributed to the excess accumulation of inorganic and non-biodegradable waste. The type of waste also contributes to the nutrient content of the soil, like farm waste may be high in organic matter content but low in nitrogen which ultimately results in low nitrogen content in the soil. As Martam landfill is located at the riverbank of Ranikhola, the soil in the landfill mainly consists of sandy soil. Consequently, sandy soils have a higher tendency to lose nitrogen through leaching (Regmi et al., 2022). In addition, soil salinity can also be the reason for low nitrogen content in soil (Akhtar et al., 2011). Moreover, the mean moisture content of the landfill soil was measured at 24.9%, which is relatively low for optimal microbial activity and organic matter decomposition (Rastogi et al., 2020). Additionally, low moisture content also corresponds with lower relative humidity within the soil microenvironment (Jin et al., 2024), which further limits the growth and function of nitrogen-fixing and ammonifying microbes (Parsons et al., 1993). As a result, total nitrogen content in the soil tends to be lower in drier environments.

High C/N ratio suggests slow decomposition process and hence corrective measures are to be taken in order to reverse this phenomenon and increase the rate of degradation.

#### Available phosphorus (P)

**Fig. 8:**
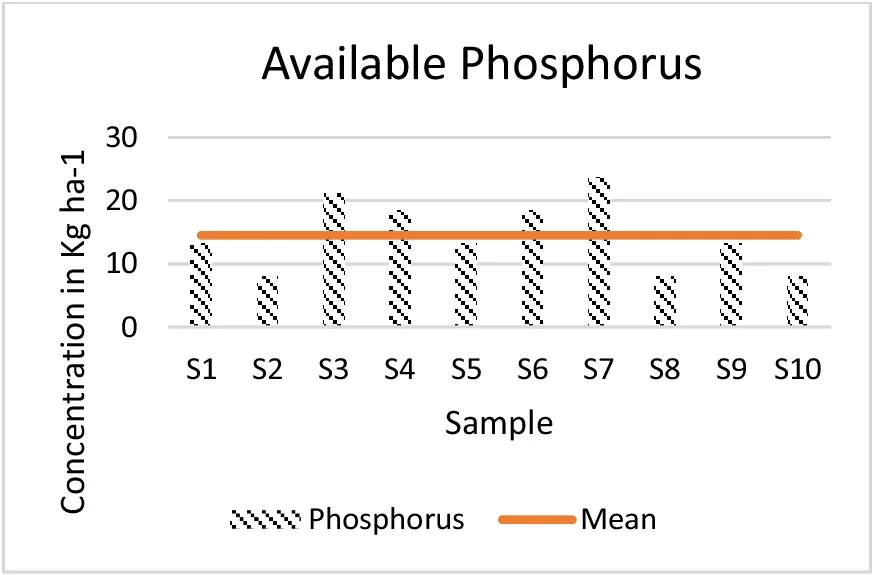
Phosphorus content in 10 soil samples, showing both individual readings and the mean. This reflects soil’s phosphorus availability

From the analyzed data, the available phosphorus content of the soil samples was found to be ranging from 8.04 to 23.632 kg ha^−1^(Fig. 8). The mean value of the available phosphorus in the soil was 14.54 kg ha^−1^ and CV was 38.86%, reflecting considerable variability among the samples. The observed value of phosphorus in the controlled site was slightly lower (11.2 kg ha^−1^). The observed values of phosphorus in both the studied dumpsite and the control site were low. The low phosphorus content may be due to the presence of non-biodegradable waste materials in higher content and low organic matter content as well as degradation of agricultural materials (Ulakpa et al., 2021). Slightly higher phosphorus content (24.61 kg ha^−1^) was reported by Agbeshie et al. (2020). However, the findings reported by Amos-Tautua et al. (2014) and Regmi et al. (2022) are in contrast to the results of the current investigation. The dumpsites had quite high levels of available phosphorus ranging from 68.22 ± 0.89 to 84.20 ± 1.02 mg kg^−1^ and 191.6 kg ha^−1^ respectively. Akintola et al. (2021) also reported similar findings. The elevated phosphorus level is linked to the leachate generated in the dump site (Deshmukh & Aher, 2017) and disposal of waste containing a high amount of organic matter (Brady & Weil, 1999; Regmi et al., 2022). Sanjana et al. (2022) also reported high levels of available phosphorus from the landfills in Vishakhapatnam, with the highest value reaching 76.4 kg ha^−1^.

#### Potassium (K)

**Fig. 9:**
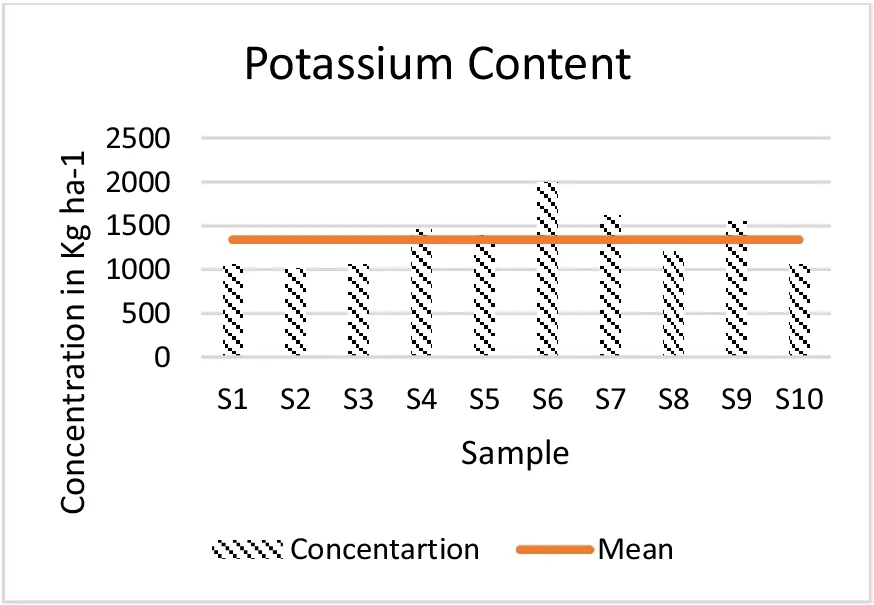
Potassium levels in 10 soil samples, including the mean value

In the present investigation, high potassium levels were observed in the dumpsite soil that ranged from 1008 to 1993.6 kg ha^−1^ (Fig. 9) with a CV of 23.99%, whereas the control site recorded a lower K value of 313.6 kg ha^−1^. This result is in conformity with those given by Regmi et al. (2022) where the elevated potassium levels (1347.7 kg ha^−1^) were reported from the dumpsite soil. Parameswari et al. (2015) also reported high levels of potassium which ranged from 188 mg kg^−1^ at 5.5 m depth to 1123 mg kg^−1^ at 2.5 m depth from the landfill located in Chennai. Olagunju et al. (2018) also reported similar findings for the value of K which ranged from 542 to 1065 ppm and conclusively reported that the soil in and around the dumpsite has high K levels due to the impact of the waste dumpsite as well as the use of N-P-K fertilizers by the farmers on the surrounding soil. This trend, however, contrasted with the report by Sanjana et al. (2022), in which the soil sample collected from different landfills in and around Vishakhapatnam city showed moderate levels of available potassium ranging from 239 to 312 kg ha^−1^ (Cheemalapalli landfill), 234 to 280 kg ha^−1^ (Arilova landfill), 215 to 312 kg ha^−1^ (Kapuluppada landfill), and 215 to 264 kg ha^−1^ (Gajuwaka landfill). Potassium, being the second most important macro element for soil and crop productivity, is not considered harmful when present in excess (Deshmukh and Aher, 2017). The reason for the observed high concentration of potassium in the soil may be due to the breakdown of waste, as composting of biodegradable waste adds traces of metallic ions including potassium, into the soil (Parameswari et al., 2015). Moreover, high potassium content may also be influenced by the low oxidation of elemental potassium in the waste particles, as well as the soil structure and the type of waste disposed of at the dumpsite (Bartkowiak et al., 2018; Gyabaah et al., 2023).

A comparative analysis of key physicochemical parameters between the present study area and several other reported landfill sites is summarized in Table 5. The table highlights variations in parameters such as pH, moisture content, total organic carbon, nitrogen, phosphorus, and potassium.

**Table 5:** Physicochemical properties of Martam landfill soil compared with data from previous studies, highlighting similarities and differences in soil characteristics

S.No	Reference	Landfill site	pH	Moisture content (%)	TOC (%)	N (kg ha^−1^)	P (kg ha^−1^)	K (kg ha^−1^)
1	Regmi et al. (2022)	Dumping sites of Baglung Municipality, Nepal	5.3 to 7.3	5.5–10.5%	**-**	119.2	191.6	1346.7
^**2**^	Akintola et al. (2021)	Lapite dumpsite, Ibadan City, Nigeria	7.35	35.22%	**-**	-	-	-
^**3**^	Sanjana et al. (2022)	Visakhapatnam dumpsites	-	**-**	-	218.3- 248	46.3–66.5	246.3–267.8
^**4**^	Ulakpa et al. (2021)	Power-line dumpsite, Boji-BojiOwa, Delta State	6.22 ± 0.06 to 7.97 ± 0.04	-	0.05 ± 0.00–0.64 ± 0.03	**-**	-	-
^**5**^	Deshmukh and Aher (2017)	Sangamner City, Maharashtra	8.4 ± 0.23	-	4.2 ± 3.83	180.9 ± 71.61	40.3 ± 18.46	167.5 ± 71.49
^**6**^	Jilani and Rashid (2020)	Karachi Metropolis	7.95	4.48	3.55	-	-	-
^**6**^	Singh and Thakur (2024)	Himachal Pradesh	6.61 ± 0.06	-	1.03 ± 0.14	348.99 ± 26.21	221.57 ± 22.97	745.35 ± 138.83
^**7**^	Raman and Narayanan (2008)	Pallavaram solid waste landfill, Chennai	6.30–7.00	8.42–9.64	**-**	-	-	-
^**8**^	Present study	Martam Landfill, East Sikkim	7.74	24.9%	1.9%	59.89	14.54	1338.4

### Statistical analysis

Descriptive statistics for six physicochemical parameters across ten landfill soil samples are presented in Table 4. Skewness values ranged from −0.503 to 0.869, indicating that most parameters exhibited near-symmetrical distributions. Potassium showed moderate positive skew (0.869), suggesting a right-tailed distribution, while moisture content and pH showed mild negative skewness (−0.503 and −0.492, respectively). Kurtosis values ranged from −1.644 to 0.241, indicating predominantly platykurtic distributions (flatter than normal), with one parameter close to mesokurtic (0.241). Overall, the data suggest approximate normality without major outliers, supporting the cautious use of parametric methods given the limited sample size (*n* = 10). Pearson’s correlation coefficients (Table 6) were used to examine associations among soil parameters. A significant positive correlation was observed between total organic carbon (TOC) and moisture content (*r* = 0.749, *p* = 0.013), suggesting co-variation likely linked to organic waste input. Moderate positive correlations were also noted between TOC and phosphorus (*r* = 0.607, *p* = 0.063) and between phosphorus and potassium (*r* = 0.528, *p* = 0.117), though these were not statistically significant. A moderate negative correlation between pH and total nitrogen (*r* = −0.606, *p* = 0.063) may indicate nitrogen loss through volatilization under alkaline conditions.

**Table 6:** Pearson’s correlation coefficients among six soil parameters across 10 samples

Correlations							
		pH	MC	TOC	TN	P	K
pH	Pearson’s correlation	1	.362	.614	−.606	.077	.218
	Sig. (2-tailed)		.304	.059	.063	.832	.545
	*N*	10	10	10	10	10	10
MC	Pearson’s correlation	.362	1	.749*	−.395	.506	.142
	Sig. (2-tailed)	.304		.013	.259	.136	.695
	*N*	10	10	10	10	10	10
TOC	Pearson’s correlation	.614	.749*	1	−.355	.607	.359
	Sig. (2-tailed)	.059	.013		.314	.063	.309
	*N*	10	10	10	10	10	10
TN	Pearson’s correlation	−.606	−.395	−.355	1	−.094	−.268
	Sig. (2-tailed)	.063	.259	.314		.797	.453
	*N*	10	10	10	10	10	10
P	Pearson’s correlation	.077	.506	.607	−.094	1	.528
	Sig. (2-tailed)	.832	.136	.063	.797		.117
	*N*	10	10	10	10	10	10
K	Pearson’s correlation	.218	.142	.359	−.268	.528	1
	Sig. (2-tailed)	.545	.695	.309	.453	.117	
	*N*	10	10	10	10	10	10

*Correlation is significant at the 0.05 level (2-tailed)

Abbreviations: *MC*, moisture content; *TOC*, total organic carbon; *TN*, total nitrogen; *P*, phosphorus; *K*, potassium

**Fig. 10:**
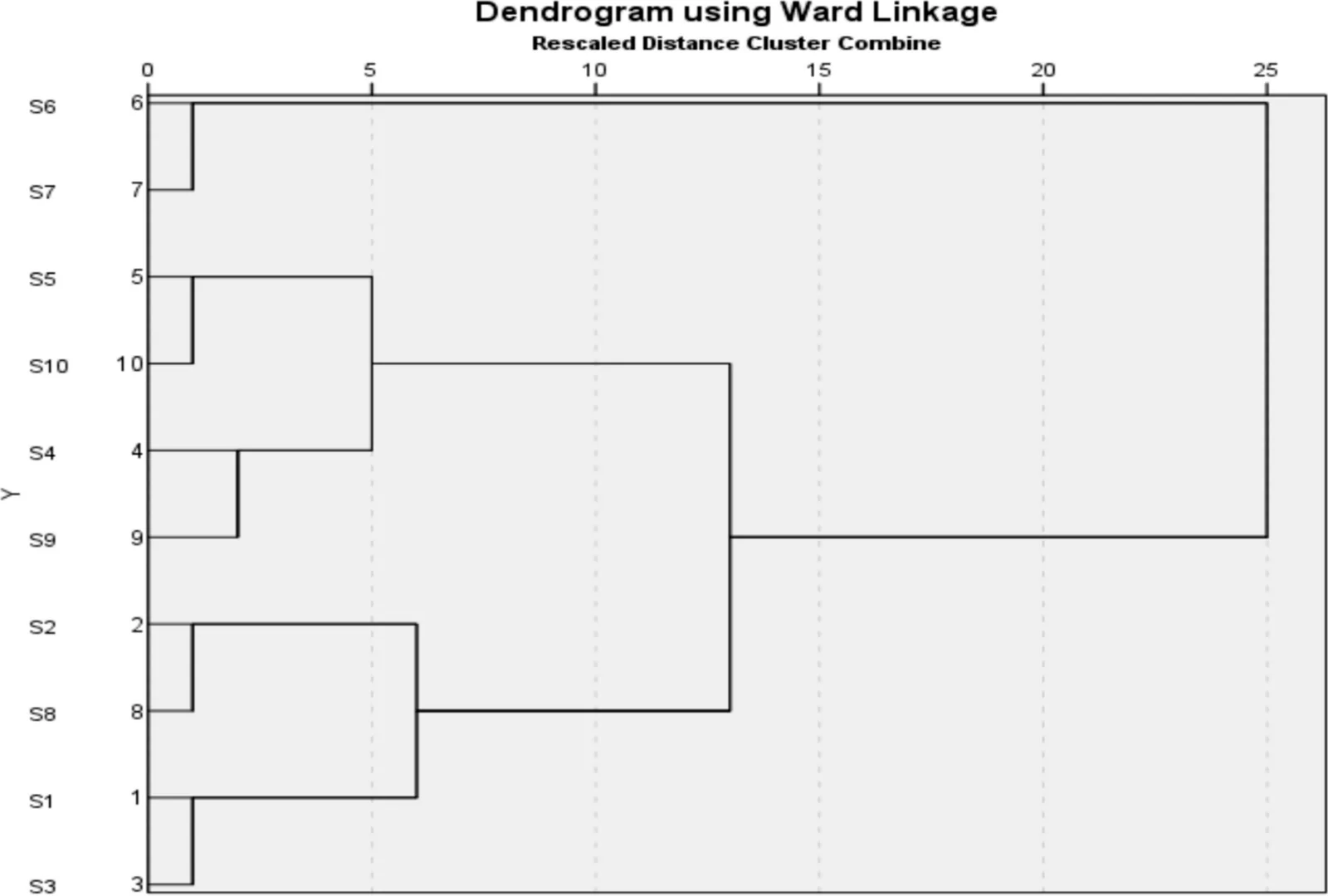
Dendrogram representing the hierarchical clustering of 10 soil samples based on six soil parameters using Ward’s linkage method

To explore sample similarity, hierarchical cluster analysis (HCA) was performed using Ward’s linkage and Euclidean distance. Examination of the agglomeration schedule (Table 7) revealed a marked increase in coefficients between stages 8 and 9, supporting the selection of three clusters. The dendrogram (Fig. 10) grouped samples S1, S2, S3, and S8 in one cluster, S4, S5, S9, and S10 in a second, and S6 and S7 in a distinct third cluster. These groupings reflect heterogeneity in soil properties—such as pH, nutrient levels, and moisture—likely influenced by varying waste composition and degrees of contamination across the landfill. Cluster-based grouping of soil samples is valuable for landfill management, as it highlights zones with comparable contamination profiles, enabling targeted remediation, optimized monitoring, and efficient resource allocation. Such an approach strengthens environmental risk assessment and supports sustainable management of landfill sites.

**Table 7:** Agglomeration schedule showing the stages merging, cluster pairs combined at each stage, and their respective linkage coefficients

Agglomeration schedule						
Stage	Cluster combined		Coefficients	Stage cluster first appears		Next stage
	Cluster 1	Cluster 2		Cluster 1	Cluster 2	
1	6	7	1.738	0	0	9
2	5	10	3.704	0	0	6
3	2	8	6.082	0	0	7
4	1	3	8.517	0	0	7
5	4	9	11.550	0	0	6
6	4	5	16.700	5	2	8
7	1	2	22.723	4	3	8
8	1	4	33.708	7	6	9
9	1	6	54.000	8	1	0

### Broader implications and actionable insights

The quantitative findings from the Martam landfill site offer a critical, data-driven foundation for designing targeted waste management and remediation strategies specifically for the fragile and rapidly urbanizing Himalayan ecosystem.

The prevalence of *Bacillus* and *Pseudomonas* suggests that the landfill functions as a natural enrichment site for waste-degrading microbes. These native strains can be developed into a specialized microbial inoculum to speed up the natural degradation process and improve compost quality. The soil nutrient patterns at the Martam landfill, characterized by low organic carbon and nitrogen but elevated potassium, serve as indicators of long-term waste deposition and provide direction for waste-processing strategies. Such insights can inform composting strategies, allowing the potassium-rich but nitrogen-poor organic fraction of MSW to be co-composted with nitrogen-rich materials like sewage slurry or poultry manure to produce balanced, high-quality compost.

The trends identified in this study offer several actionable insights for waste management in the Himalayan context:

1. Integrating microbial or bioengineered remediation into existing waste management frameworks to accelerate degradation of plastics and organic waste.
2. Addressing nutrient imbalances through routine soil monitoring and implementing remediation measures such as phytoremediation, organic amendments, and engineered soil covers to stabilize nutrient cycling and minimize leachate contamination of downstream water sources.
3. Diverting biodegradable waste away from landfills toward decentralized composting and anaerobic digestion units, which are highly feasible in Himalayan settlements due to smaller waste volumes.

Despite the promising microbial capacity for degradation, relying solely on natural attenuation is inadequate to manage the escalating load of plastic and municipal waste. A waste management strategy that combines microbial bioremediation, segregation at source, composting, anaerobic digestion, and the adoption of eco-friendly materials is essential to reduce long-term environmental risks. Strengthening waste-handling infrastructure, fostering community participation, and investing in research on local microbial resources will be critical for sustainable waste governance. Ultimately, the observed microbial and soil patterns from Martam provide a scientific foundation for designing region-specific remediation technologies and evidence-based policies that can enhance ecological resilience in fragile mountain ecosystems.

### Limitations and future directions

While this research offers insights into the bacterial population of the landfill soil, its findings must be interpreted considering certain constraints. The identification of bacterial isolates was restricted to traditional phenotypic methods. The absence of molecular genetic analysis prevents definitive species-level identification and obscures the genetic potential of these isolates. Although the presence of *Bacillus* and *Pseudomonas* suggests potential for bioremediation, we did not experimentally test the degradation capabilities of these isolates under controlled or field conditions. Furthermore, the study did not include a characterization of soil texture (particle size distribution). As soil type directly influences key physicochemical properties such as water-holding capacity, nutrient retention, and contaminant mobility, its absence limits a more comprehensive interpretation of the observed microbial and physicochemical results.

Future work should focus on laboratory and field-based validation of microbial potential for plastic and waste degradation, as well as controlled trials to test balanced nutrient composting and engineered landfill strategies. Expanding the scope of microbial, physicochemical, and soil texture analyses will provide stronger, experimentally supported evidence to guide sustainable waste management in the Himalayan region.

## Conclusion

The MSW samples collected from Martam, a landfill site serving a rapidly urbanizing Himalayan city in Sikkim, revealed a diverse microbial community comprising both gram-positive and gram-negative bacteria, with *Bacillus* species being the most prevalent. The isolation of *Bacillus* and *Pseudomonas* species—microorganisms known for their capacity to biodegrade low-density polyethylene (LDPE)—suggests the landfill’s potential as a natural bioreactor for plastic degradation. Soil analysis showed pH ranging from 7.38 to 8.02 (mean 7.74 ± 0.19), moisture content from 18.02 to 30.27% (mean 24.9 ± 4.04%), total organic carbon 0.88–3.18% (mean 1.9 ± 0.81%), total nitrogen 25.08–94.08 kg ha^−1^ (mean 59.89 ± 25.06 kg ha^−1^), available phosphorus 8.04–23.63 kg ha^−1^ (mean 14.54 ± 5.65 kg ha^−1^), and potassium 1008–1993.6 kg ha^−1^ (mean 1338.4 ± 321.1 kg ha^−1^). Compared to the control site, the landfill soils showed reduced organic carbon and nitrogen but higher potassium and variable phosphorus levels, indicating both the nutrient-enriching and nutrient-depleting effects of prolonged waste deposition.

While the microbial potential for biodegradation is promising, natural attenuation alone is insufficient. Integrated waste management including composting, anaerobic digestion, and eco-friendly materials is essential. Strengthening research, infrastructure, and public awareness will be critical to reducing the long-term environmental impact of plastic and municipal waste.

## Data Availability

No datasets were generated or analysed during the current study.

## Declarations

All authors have read, understood, and have complied as applicable with the statement on “Ethical responsibilities of Authors” as found in the Instructions for Authors.
